# Bridging Kidney Organoid Innovation and Regenerative Medicine: Current Advances and Future Directions

**DOI:** 10.1111/cpr.70228

**Published:** 2026-05-15

**Authors:** Bohong Guo, Jingyuan Zhang, Xuening Fang, Yangyang Liu, Yueyang Deng, Yuxin Xue, Zhengxi Wang, Xiaotao Dong, Mengqi Jia, Xiaodong Li

**Affiliations:** ^1^ Department of Urology, The First Affiliated Hospital Henan University Kaifeng China; ^2^ Laboratory of Receptor Gene Regulation and Drug Discovery, School of Basic Medical Sciences Henan University Kaifeng China; ^3^ Guangdong Provincial Key Laboratory of Medical Immunology and Molecular Diagnostics, The First Dongguan Affiliated Hospital, School of Medical Technology Guangdong Medical University Guangzhou China

**Keywords:** bioengineering, epigenetic regulation, kidney organoids, regenerative medicine, signalling pathways, single‐cell transcriptomics

## Abstract

Chronic kidney disease (CKD) has emerged as a critical public health challenge worldwide, and organ donor shortages underscore the urgent need for alternative therapeutic strategies. Advances in stem cell technologies have enabled the generation of kidney organoids, providing innovative platforms to model renal development, investigate disease mechanisms, support drug discovery, and explore applications in regenerative medicine. Yet, limitations such as immature tissue architecture, insufficient vascularisation, and unaddressed safety concerns still hinder their translation into regenerative medicine. In this review, we summarise the fundamentals of kidney development, current differentiation approaches, and the signalling and epigenetic mechanisms underlying organoid lineage specification. We further highlight the roles of bioengineering innovations and single‐cell transcriptomics in establishing evaluation frameworks and enhancing structural complexity. We finally emphasise that existing optimisation frameworks, primarily focused on improving differentiation efficiency and enforcing relatively restricted lineage specification, may prove inadequate for bridging the gap to clinical translation. Instead, the most promising paradigm shift involves the convergence of bioengineering modulation and high‐resolution functional assessment to facilitate the synchronised advancement of organoid complexity and physiological utility.

## Graphical abstract

This review outlines kidney organoid differentiation strategies, key developmental mechanisms, and engineering and multi‐omics approaches driving their progress toward regenerative medicine.

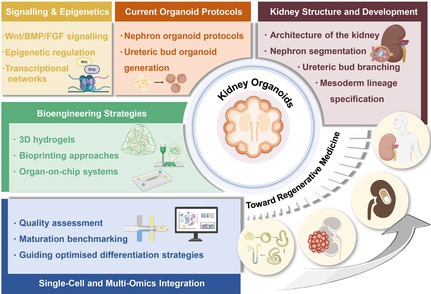

## 1 Introduction

The kidney is essential for maintaining fluid and electrolyte homeostasis and eliminating metabolic waste. However, in reality, more than 850 million individuals suffer from chronic kidney disease (CKD), which eventually progresses to end‐stage renal disease [1]. Although organ transplantation remains the most effective treatment option, its clinical application is significantly limited by the scarcity of available donors [2]. Recent progress in stem cell biology has opened new possibilities [3], positioning kidney organoids as an attractive strategy to recapitulate renal development and function in vitro [4, 5].

A decade of progress has enabled the in vitro generation of both nephron organoids and ureteric bud organoids, modelling the kidney's filtering and collecting systems, respectively. Multiple research teams have established reliable differentiation protocols and continuously optimised them [6, 7, 8, 9, 10]. These organoids have already been applied to genetic disease modelling, drug screening, and nephrotoxicity testing, indicating their strong translational potential [11, 12].

Nonetheless, considerable challenges persist, including inadequate maturation, limited vascular integration, and uncertain safety for therapeutic use. Bioengineering strategies, including biomimetic extracellular matrices, 3D bioprinting, and organ‐on‐chip platforms, offer promising avenues for enhancing functionality [13, 14]. Complementarily, as a key tool for evaluating the development status of organoids, single‐cell transcriptomics can analyse differences in cellular composition and identify uncharacterised developmental features [15, 16]. Further combination with multi‐omics approaches is expected to hasten optimisation of differentiation strategies.

In this review, we aim to integrate insights from kidney developmental biology with current strategies for kidney organoid generation, with a particular focus on the signalling pathways and epigenetic mechanisms that govern lineage specification. By considering advances in bioengineering together with single‐cell level evaluation strategies, we further seek to clarify how the structural and molecular maturity of kidney organoids can be more systematically and rigorously assessed and improved.

## 2 The Rise and Evolution of Kidney Organoids

Kidney organoids are 3D in vitro systems derived from induced pluripotent stem cells (iPSCs), embryonic stem cells (ESCs), or adult stem cells that recapitulate key structural and functional features of the kidney, with applications in disease modelling, drug screening, and regenerative medicine [17, 18, 19, 20]. In essence, the emergence of kidney organoids is driven by both clinical necessity and advances in developmental biology: the former brings to the fore the urgent need for renal replacement and repair, while the latter provides the theoretical and technical framework for in vitro construction.

### 2.1 Unmet Clinical Needs

The kidney maintains systemic homeostasis by filtering blood and regulating water‐electrolyte balance via urine formation. The nephron serves as the fundamental functional unit, comprising a renal corpuscle and a sequential tubular system that processes filtrate into final urine [21]. Chronic kidney disease (CKD), defined by structural or functional abnormalities of the kidney persisting for more than 3 months [1], is a major global health burden and ranks among the leading causes of mortality and morbidity worldwide [22]. Current treatments remain limited: according to the Global Burden of Disease Study 2023, the global prevalence of kidney transplant recipients reached approximately 1.02 million, whereas 3.57 million patients were maintanined on dialysis [23], underscoring a substantial gap between supply and demand. Dialysis fails to fully replicate essential renal functions and is associated with high costs and reduced quality of life [24]. Although alternative strategies such as xenotransplantation and interspecies organogenesis have been explored [25, 26, 27], immunological incompatibility and uncertain long‐term safety remain major obstacles. Thus, developing novel in vitro models that mimic renal structure and function is an urgent scientific and clinical priority. In this context, kidney organoids are being developed to address these needs and have begun to show progress in areas such as mechanistic studies of CKD and the exploration of cell‐based therapeutic strategies [28, 29, 30].

### 2.2 Developmental Basis for Kidney Organoids Construction

**FIGURE 1:**
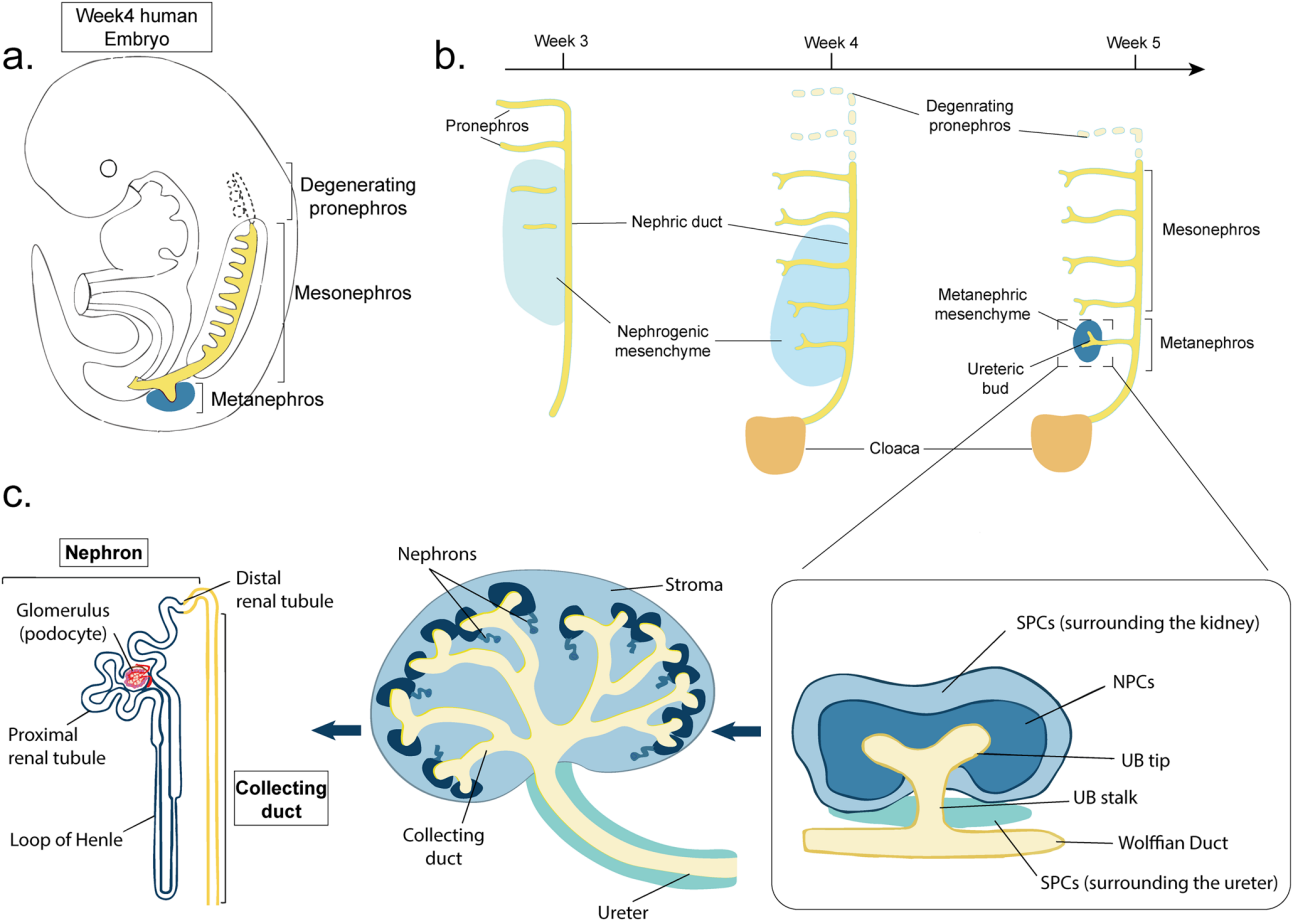
Overview of the human kidney development process. (a) Human kidney development at 4 weeks of gestation. (b) Human kidney formation proceeds through three successive stages: Pronephros, Mesonephros, and Metanephros, with the permanent kidney ultimately establishing from the metanephros. (c) Mammalian renal development originates from the metanephros. This embryonic structure comprises two key components: the branching ureteric bud (UB) and the metanephric mesenchyme (MM). Within the MM, nephron progenitor cells (NPCs) and stromal progenitor cells (SPCs) reside as distinct precursor populations. NPCs undergo differentiation to form nephrons, specifically glomeruli and renal tubules, while adjacent SPCs generate renal stromal tissue and specialised functional cells. Notably, SPCs enveloping the UB stalk represent a separate lineage, committed to ureteral stroma formation. Concurrently, the UB undergoes iterative branching, ultimately differentiating into collecting ducts and ureteral epithelium.

Advances in kidney organoid research are rooted in an improved understanding of kidney development. Mammalian kidney development originates from the intermediate mesoderm and follows a highly coordinated and evolutionarily conserved process [31, 32], ultimately giving rise to the metanephros, the definitive kidney (Figure 1). This process is driven by reciprocal interactions between two key progenitor lineages: the ureteric bud (UB), derived from the Wolffian duct, and the metanephric mesenchyme (MM), which contains nephron progenitor cells (NPCs) and stromal progenitor cells (SPCs) [33, 34]. Their dynamic interplay governs branching morphogenesis, nephron formation, and the establishment of higher‐order kidney architecture [35, 36]. In addition, endothelial progenitor cells contribute to vascularisation and functional maturation [37].

Studies in both mouse and human embryonic kidney development have confirmed the distinct embryonic origins of the UB and MM. The UB originates from the anterior intermediate mesoderm (AIM), whereas the MM arises from the posterior intermediate mesoderm (PIM) [5]. Research on kidney developmental processes not only deepens insights into kidney organogenesis but also delineates the pathways for in vitro induction of these two renal lineages.

## 3 Major Protocols for Inducing Kidney Organoid Differentiation

Over the past decade, kidney organoid technology has progressed from concept to reality [38, 39, 40, 41, 42]. Established differentiation protocols can be broadly categorised into two types: nephron organoids and ureteric bud organoids. Building upon this, recent studies have further focused on the in vitro expansion and potential applications of key progenitor cells, thereby driving the transition of organoid systems from structural reconstruction toward functional and translational dimensions.

### 3.1 Differentiation Protocols for Nephron Organoids

The canonical nephron organoid differentiation protocol involves long‐term Wnt signalling stimulation to induce PIM, which serves as the developmental origin of NPCs. Followed by stimulation with Wnt and fibroblast growth factor (FGF) signals, NPCs differentiate into nephrons. The pivotal breakthrough originated from the insight by Taguchi et al. that the intermediate mesoderm exhibits distinct spatiotemporal differentiation fates [5]. Based on an embryoid body culture system, they first induced posterior nascent mesoderm into MM in vitro by lowering the Wnt agonist concentration while adding retinoic acid (RA) and FGF9, before advancing it further from an earlier stage. The resulting MM cells derived from hiPSCs were then co‐cultured with mouse embryonic spinal cord, which acts as an inducer of mesenchymal‐to‐epithelial transition (MET). This process yielded glomeruli containing podocytes and renal tubules, thus generating 3D structures resembling nephrons.

Following this study, Takasato et al. and Morizane et al. independently established systematic and robust protocols for generating nephron organoids from human pluripotent stem cells [6, 7, 43, 44]. It is noteworthy that the Takasato protocol utilised CHIR99021 (a Wnt agonist) to demonstrate the critical influence of Wnt signalling exposure duration on the anterior–posterior patterning of the intermediate mesoderm. The organoids generated by this method not only contained segmented, nephron‐like structures but also comprised multiple lineages, including ureteric epithelium, vascular endothelial cells, and stromal cells. Alternatively, the Morizane protocol specifically targeted the induction of the late primitive streak using a 2D monolayer culture system, achieving a markedly high differentiation efficiency of 75%–92% for NPCs.

Subsequent research groups established various kidney organoid differentiation systems based on this foundation [8, 45, 46], most involving factors like CHIR99021, FGF9, B27 serum‐free supplement, and RA. Together, these factors constitute the core methodological framework for current nephron organoids differentiation.

### 3.2 Differentiation Protocols for Ureteric Bud Organoids

Directed differentiation protocols for ureteric bud organoids emerged more recently, with protocols focusing on precise biochemical control to obtain AIM. Taguchi and colleagues identified the optimal window for using a Wnt agonist to induce AIM, noting that the required duration of activation was shorter than that needed to induce PIM [9]. This finding further corroborated the observations made earlier by Takasato et al. Subsequently, by applying stage‐specific regimens of small molecules, which included a BMP inhibitor, RA, and glial cell line‐derived neurotrophic factor (GDNF), they successfully generated UB organoids with branching capability.

**FIGURE 2:**
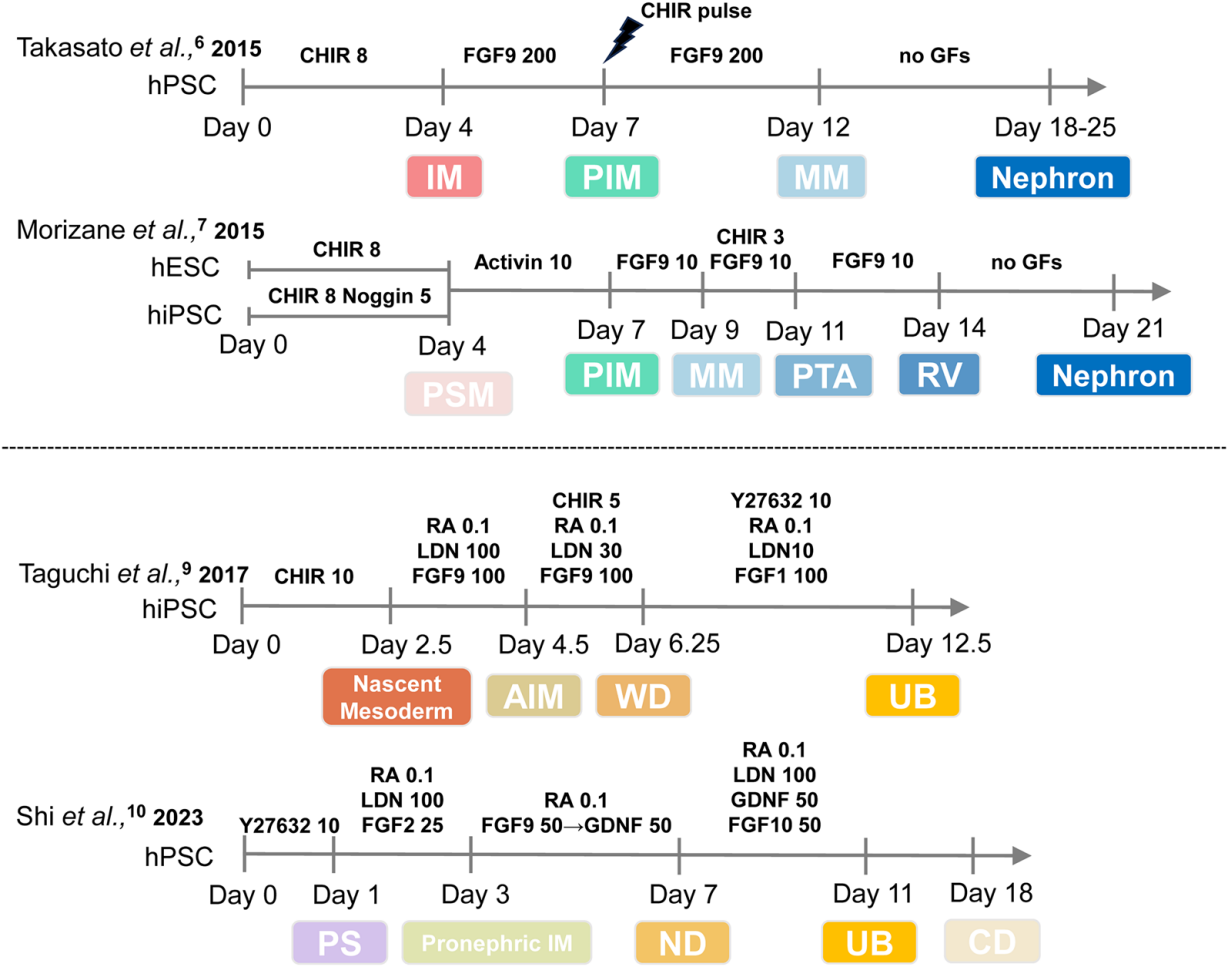
The differentiation protocols into kidney organoids from hPSCs. Specific steps in the temporal progression of different differentiation protocols for nephron organoids and ureteric bud organoids. The small molecules and growth factors added at each differentiation step and their concentrations are indicated as well as the corresponding developmental stages. The following numbers indicate concentrations: μM for CHIR, RA, Y27632; ng/ml for FGF9, Noggin, Activin, FGF1, FGF2, GDNF and FGF10; nM for LDN. (CHIR) CHIR99021 (Wnt pathway agonist); (FGF9) fibroblast growth factor 9; (no GFs) no growth factors added to basal medium; (RA) retinoic acid; (LDN) LDN193189; (FGF1) fibroblast growth factor 1; (FGF2) fibroblast growth factor 2; (GDNF) glial cell line‐derived neurotrophic factor; (FGF10) fibroblast growth factor 10.

Further research has aimed at boosting the budding and branching capacity of the resulting UB organoids. In these structures, the tip cells of the branches give rise to new tip cells and stalk cells. These progenitor populations then differentiate into CD cells, which are crucial for the organoids' functionality. Zeng et al. reported a culture system capable of expanding UB organoids with 3D branching structures in vitro and driving their differentiation into CD‐like organoids exhibiting greater functional maturity [47]. In a related development, Shi et al. proposed an optimised protocol that generates UB organoids which, upon differentiation, yield CD‐type organoids comprising over 95% authentic CD cell types [10] (Figure 2).

### 3.3 Strategies for Nephron Progenitor Cell Expansion and Related Applications

Building on existing differentiation systems, research in recent years has not only focused on structural construction but also on the optimisation of key progenitor cell resources, particularly the efficient acquisition and long‐term maintenance of NPCs (Table 1). As the core progenitor population in kidney development, NPCs possess the potential to differentiate into multiple nephron cell types and serve as the critical foundation for driving organoids toward functionalisation and application.

**TABLE 1:** Protocols for the differentiation of nephron organoids.

Protocol	Key signalling modulation	Developmental stage targeted	Mark gene at the NPC stage	Major cell types in the organoids
Taguchi et al. [5]	Wnt↓; Activin/Nodal↑; BMP ↑	Posterior nascent mesoderm → posterior intermediate mesoderm → metanephric mesenchyme	PAX2^+^, SIX2^+^, SALL1^+^, WT1^+^	WT1^+^/NPHS1^+^ glomeruli; CDH6^+^ proximal tubule structures; CDH1^+^ distal tubule structures
Takasato et al. [6]	Wnt/β‐catenin↑; RA ↑; FGF ↑	Intermediate mesoderm → metanephric mesenchyme	PAX2^+^/ECAD^−^	WT1^+^/NPHS1^+^ glomeruli; CDH1^+^/LTL^+^ proximal tubules; CDH1^+^ distal tubules; PAX2^+^/GATA3^+^/CDH1^+^ ureteric bud‐like cells
Morizane et al. [7]	Wnt/β‐catenin↑; Activin/Nodal↑	Late primitive streak → posterior intermediate mesoderm → metanephric mesenchyme	PAX2^+^, SIX2^+^, SALL1^+^, WT1^+^	NPHS1^+^/PODXL^+^ podocytes; LTL^+^/CDH2^+^ proximal tubules; CDH1^+^/UMOD^+^ loop of Henle; CDH1^+^ distal tubules
Recent NPC expansion systems				
Li et al. [17]	p38 MAPK↓; TGF‐β↓; BMP↓; YAP/TAZ↑	Nephron progenitor maintenance and expansion for 3 months	PAX2^+^, SIX2^+^	PODXL^+^/NPHS1^+^ glomeruli; LTL^+^/HNF4A^+^/SLC34A1^+^/SLC27A2^+^ proximal tubule; PAX2^+^/SLC12A1^+^ loop of Henle
Osafune et al. [30]	Wnt/β‐catenin↑; FGF↑; ROCK↓	100‐fold expansion of nephron progenitors within two passages	OSR1^+^, SIX2^+^	NPHS1^+^/PODXL^+^/WT1^+^ glomeruli; LTL^+^/CDH1^−^ proximal tubules; BRN1^+^/DBA^−^ loop of Henle; BRN1^+^/DBA^+^/CDH1^+^/LTL^−^ distal tubules

*Note:* Unlike earlier differentiation protocols that generate nephron progenitors directly from pluripotent stem cells, expandable NPC systems establish a stable progenitor population that can be clonally expanded prior to organoid differentiation, enabling scalable organoid production. This feature is particularly critical for regenerative medicine, as it offers a renewable and well‐defined cell source for cell‐based therapies, while simultaneously improving the consistency and scalability of disease modelling and drug screening applications.

Representative efforts in this direction have led to the development of advanced NPC expansion systems. Li et al. developed the hNPSR‐v2 culture system enabling sustained expansion of induced NPCs (iNPCs) from human embryonic stem cells, which were further applied to small‐molecule screening in a polycystic kidney disease (PKD) model, successfully identifying cyst‐suppressing compounds, recapitulating known drugs such as metformin, and revealing a novel candidate (PTC‐209), thereby demonstrating their translational potential as a drug discovery platform [17]. Separately, Osafune et al. established the CFY medium, a suspension culture system containing CHIR99021, FGF9, and Y27632, which mitigates the loss of differentiation potential during NPC passaging [30]. Importantly, transplantation of expanded NPCs into chemically induced CKD mouse models attenuated renal injury progression and showed potential in delaying renal aging [30], highlighting their promise for cell‐based therapies in kidney diseases.

Still, nephron organoids constructed based on NPCs lack a complete excretory pathway structure, and their overall maturity remains limited. These factors, to some extent, constrain their further functional simulation capabilities and clinical translational potential.

## 4 Molecular and Epigenetic Mechanisms Guiding Kidney Organoid Differentiation

The in vitro construction of kidney organoids is essentially a recapitulation of the multi‐level regulatory networks present during embryonic development. Within this process, established developmental signalling pathways and epigenetic regulation act synergistically to determine the direction, timing, and efficiency of lineage differentiation.

### 4.1 Molecular Signalling Mechanisms in Kidney Organoid Differentiation

From a developmental perspective, constructing kidney organoids centers on three critical nodes: first, induction of hPSCs to mesoderm; second, further induction along the anterior–posterior axis to form AIM and PIM; finally, derivation of ureteric bud organoids and nephron organoids from AIM and PIM, respectively [5, 48, 49].

IM specification originates from the PS, more precisely, from late PS, driven primarily by Wnt and BMP4 signalling [50, 51, 52, 53]. The duration of Wnt signal exposure influences the anterior–posterior fate of the mesoderm, with RA gradients also demonstrating utility [49, 54]. During the subsequent differentiation from PIM to MM, sustained FGF9 signalling is required to maintain the proliferation and ongoing differentiation of NPCs [7]. Contemporaneously, a transient Wnt signal, mimicking that derived from the UB, is essential to catalyse the MET in NPCs. Following the formation of the renal vesicle, growth factors can be withdrawn, allowing the structures to spontaneously self‐organise into sequentially connected nephron segments.

Of particular note, Lindström et al. conducted an in‐depth investigation into the molecular signals governing the establishment of the proximal‐to‐distal axis during early nephron formation [55]. They identified the combination of Wnt activation and BMP inhibition as a crucial signalling node, a finding that contributes to the generation of nephron structures that more closely mirror their in vivo counterparts.

**FIGURE 3:**
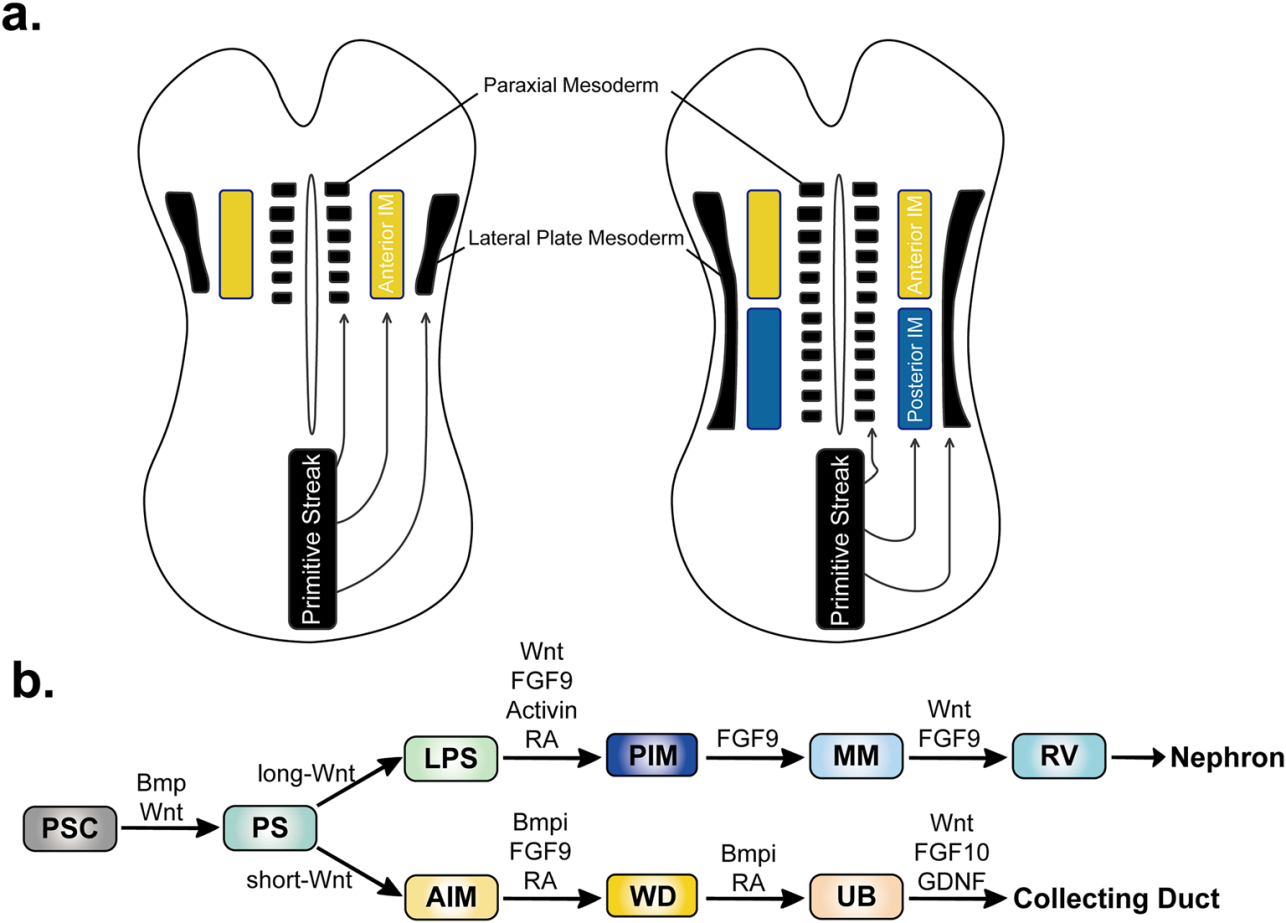
Directed differentiation of renal lineages via temporal control of intermediate mesoderm signalling programs. (a) The anterior–posterior identity of the intermediate mesoderm is determined by the timing of its migration from the primitive streak, with early migration giving rise to anterior intermediate mesoderm and late migration to posterior intermediate mesoderm. (b) Key signalling nodes involved in the differentiation of nephron organoids and ureteric bud organoids.

For the induction of UB organoids, the sustained administration of RA and a BMP inhibitor proves effective [9]. Subsequent branching morphogenesis of the UB requires activation of signals like Wnt, FGF, and Ret to develop into the urine collection system, including CD [56, 57, 58, 59] (Figure 3).

### 4.2 Epigenetic Regulatory Mechanisms in Kidney Development

Intricately, epigenetic regulation plays a crucial role in modulating renal lineage specification and differentiation, acting in concert with key developmental signalling pathways [60]. Current research on epigenetic regulation has relatively focused on NPC proliferation and nephron differentiation. It is widely recognised that global DNA hypomethylation and histone hyperacetylation adversely affect these processes [61, 62]. Among the specific DNA methylation patterns of the kidney, DNA methyltransferase 1 (DNMT1) has been shown to be indispensable for the development of functional nephron epithelial cells [63, 64]. Global DNA hypomethylation can disrupt the progenitor regulatory networks, leading to downregulation of *Wt1* and its downstream target *Wnt4*, both of which are critical for nephrogenesis initiation [62, 65]. This consequently negatively regulates Wnt signalling during the mesenchymal‐to‐epithelial transition and impedes nephron formation.

Regarding histone modifications, histone deacetylases (HDACs) participate in regulating *SIX2* expression, thereby promoting the development and differentiation of the RV into nephrons [62, 66]. More broadly, NPCs are maintained in an epigenetically repressed yet transcriptionally poised state. In undifferentiated NPCs, HDAC1/2, EZH2, and G9a mediate histone deacetylation and methylation at H3K9 and H3K27, keeping nephrogenic loci transcriptionally silent while RNA polymerase II remains paused at promoter regions. Upon activation of canonical Wnt/β‐catenin signalling, *SIX2* expression declines and nuclear accumulation of β‐catenin triggers local chromatin remodelling. This transition is characterised by increased promoter accessibility, enrichment of the activating H3K4me3 mark, and concomitant depletion of H3K27me3/EZH2, thereby releasing transcriptional repression and initiating gene programs that drive nephron differentiation and MET [65, 67]. For the UB, HDAC1 and HDAC2 have been demonstrated to be essential for UB cell growth, survival, and branching morphogenesis [68]. Additionally, microRNAs play significant roles in UB branching morphogenesis [69, 70]. A thorough understanding of epigenetic regulation during kidney development provides a clear framework, which may help overcome certain barriers encountered in kidney organoid differentiation.

Collectively, epigenetic regulation, by coordinating chromatin states and transcriptional activity, works in conjunction with molecular signalling pathways to constitute the critical regulatory foundation for kidney organoid differentiation.

## 5 Bioengineering and Analytical Strategies in Kidney Organoids

A prevailing recognition in current kidney organoid research is that their structural integrity and functional maturity remain significantly suboptimal, falling short of the requirements for clinical application. Beyond the optimisation of biochemical induction protocols, the integration of bioengineering technologies provides essential strategies to enhance structural complexity and functional performance. Concurrently, the advancement of single‐cell sequencing and multi‐omics analysis has furnished critical tools for the systematic evaluation and refinement of organoid systems. The convergence of these two fields is driving the evolution of kidney organoids from merely recapitulating structure to achieving true functional reconstruction.

### 5.1 Bioengineering Strategies for Enhancing Organoid Maturation

To surmount the organisational limitations of conventional culture systems, bioengineering employs engineered construction to introduce spatial control, integration of multiple cell types, and physical environmental cues, thereby augmenting tissue complexity. These strategies include biomimetic matrices, 3D bioprinting, and organ‐on‐a‐chip platforms (Table 2), which aim to recreate key structural and mechanical features of the native microenvironment and thereby promote the maturation and physiological relevance of kidney organoids [71, 72, 73, 74, 75].

**TABLE 2:** Applications of bioengineering strategies for kidney organoid development.

Engineering strategy	Key principle	Major advantages	Typical applications	Representative technologies	Limitations
Hydrogel matrices	ECM mimicry and tunable stiffness	Biomimetic microenvironment; supports differentiation	Lineage specification; nephrogenesis studies	Hyaluronic acid (HA)‐based hydrogels; Gelatin Methacryloyl (GelMA) hydrogels; Self‐assembling peptide hydrogels (SAPHs)	Batch variability affects reproducibility; Lack of perfusion limits maturation
3D Bioprinting/biofabrication	Spatially controlled cell deposition	Precise architecture; scalable fabrication	Tissue patterning; organoid assembly	Stereolithography; Digital light processing (DLP); 3D bioprinting of cellular bioinks	Limited resolution for fine structures; High cost and technical barriers
Microfluidic organ‐on‐chip	Controlled perfusion and shear stress	Promotes vascularisation and functional maturation	Injury modelling; drug nephrotoxicity testing	Kidney organoid‐on‐chip; Proximal tubule‐on‐chip; Glomerulus‐on‐chip	Technical complexity and low scalability; Standardisation remains challenging

Among biomimetic matrices, hydrogels are extensively studied. Their customisable viscosity and mechanical properties allow the simulation of the natural extracellular matrix (ECM) environment [76, 77, 78]. Crean et al. cultured hiPSC‐derived kidney organoids in fully synthetic self‐assembling peptide hydrogels (SAPHs) with adjustable stiffness [79]. This resulted in podocytes exhibiting a more mature gene expression profile and significantly reduced off‐target cell types, confirming the influence of the biophysical environment on cell fate. Further single‐cell transcriptomic analysis revealed that, compared with organoids cultured in the softer Alpha4 matrix, organoids derived from Alpha5 under stiffer matrix conditions exhibited increased expression of mature podocyte markers, including NPHS2, ANXA1, and PODXL, indicating enhanced podocyte maturation. These findings suggest that matrix mechanical properties can modulate differentiation efficiency and organoid maturation by regulating progenitor cell states and lineage bias. Similarly, viscoelastic alginate hydrogels enhance nephron structural complexity toward in vivo‐like architectures [80], while decellularised porcine or human kidney extracellular matrix further recapitulates native composition and supports vascular structure formation [81, 82].

3D bioprinting is a novel technology using computer‐aided design to construct complex biological structures, enabling spatial control over multiple cell types and bioactive factors [83, 84, 85]. Lawlor et al. utilised extrusion‐based bioprinting to generate kidney organoids with functional proximal tubule segments [86]. Subsequent scRNA‐seq analyses further revealed enhanced nephron maturation and an increased number of glomerular structures in these engineered organoids. 3D bioprinting facilitated large‐scale production for high‐throughput screening, enhancing the applicability and reproducibility of kidney organoids for disease modelling and drug screening.

Beyond diverse cell types and complex matrix environments, kidney tissue is also subject to substantial fluid shear stress and mechanical tension as a result of its role in blood filtration, which are important physical environmental cues [87]. Microfluidic chips simulate in vivo fluid flow conditions [88, 89], thereby promoting the formation of endothelial cell networks within kidney organoids [90]. Homan et al. experimentally demonstrated that elevated shear stress induces VEGF upregulation, which increases endothelial progenitors and promotes tubular epithelial maturation through vascular‐tubular interactions [91].

Overall, within engineered systems, bioengineering strategies reshape the local microenvironment, primarily composed of endothelial cells, stromal cells, and extracellular matrix, by introducing these cell types and regulating their spatial arrangements, thereby positioning these cues as critical regulators of lineage specification and maturation [92, 93]. Simultaneously, it leverages the mechanical properties of biomaterials, such as stiffness and viscoelasticity, to further modulate differentiation efficiency [94]. The intrinsic coupling of bioengineering with microenvironmental regulation establishes a hierarchical structure–environment function framework, providing the substantial foundation for the progressive maturation of kidney organoids.

### 5.2 Applications Enabled by Engineered Kidney Organoids

Supported by bioengineering strategies, the utility of kidney organoids has expanded toward clinical translation, particularly in drug screening and regenerative medicine research.

In the realm of drug screening, engineered organoids more closely approximate physiological states due to their enhanced structure and function, thereby increasing the accuracy of drug response assessments [95, 96, 97]. In the context of renal cell carcinoma (RCC), drug testing in patient‐derived RCC organoids produced by 3D bioprinting revealed distinct inter‐patient heterogeneity in treatment responses and accurately predicted drug sensitivity [98], highlighting its potential for personalised therapeutic stratification. Furthermore, the implementation of a “proximal tubule‐on‐a‐chip” (PToC) has been shown to markedly upregulate OAT1/3 and OCT2 expression in kidney organoids, thereby excelling in modelling physiological drug transport and nephrotoxicity for compounds like adefovir and cisplatin [99]. Integration with patient‐derived iPSCs further paves the way for applications in personalised medicine.

In regenerative medicine exploration, engineering strategies offer new possibilities for constructing functional tissue units. For example, organoid structures created through 3D bioprinting could theoretically serve as nephron patches to increase the number of functional nephrons [91]. Additionally, nephron progenitor cell‐derived molecules induced by hydrogel microenvironments (iNPC‐SMs) have demonstrated therapeutic potential as a cell‐free strategy in cisplatin‐induced acute kidney injury models [29].

Despite these advancements extending the boundaries of organoid applications, challenges remain regarding large scale production, standardised control, and long‐term functional stability. These issues continue to limit their clinical translation to a certain extent.

### 5.3 Utilisation of Single‐Cell Sequencing for the Evaluation of Kidney Organoids

A critical unresolved issue in the kidney organoid field is establishing a relatively standardised assessment system to evaluate organoid quality and functional maturity [100]. The high cellular diversity and inter‐individual heterogeneity of kidney tissue mean there is still a lack of clear consensus on the number and distribution range of various renal cell types [101, 102], which poses a major obstacle in both delineating developmental trajectories and benchmarking organoid fidelity [103, 104, 105]. Single‐cell RNA sequencing (scRNA‐seq) may offer a powerful solution to this issue.

scRNA‐seq enables high‐resolution transcriptomic profiling of kidney organoids, allowing unbiased identification of cell types, assessment of differentiation fidelity, and detection of off‐target populations [106, 107, 108]. This approach overcomes the limitations of bulk RNA‐seq and has been widely applied to evaluate and optimise kidney organoid differentiation strategies. Wu et al. used scRNA‐seq for the first direct comparison of Takasato and Morizane differentiation protocols, revealing the presence of non‐renal cells (primarily neuronal and muscle) in both protocols and identifying that “ureteric bud and derivatives” were actually distal tubules [15]. Moreover, Subramanian et al. employed this technology to investigate the robustness of four organoid protocols across different iPSC lines and found that mouse kidney subcapsular transplantation reduced off‐target cell formation [109]. Furthermore, Little et al. developed DevKidCC, a data analysis tool for direct comparison of different kidney organoid protocols, which is crucial for organoid evaluation [16].

In parallel, systematic comparison with healthy kidney single‐cell atlases allows precise assessment of the relative abundance or absence of key cell types (e.g., glomerular endothelial cells, principal cells, intercalated cells, immune cells) within organoids [110, 111]. For example, Combes et al. compared human kidney organoids with fetal kidney scRNA‐seq data [112], uncovering consistent key cell markers but underrepresentation of distal nephron cells in organoids. They also observed dysregulated expression of myogenic genes (e.g., MYOD1/MYOG) in some NPCs, suggesting maintenance of progenitor properties via aberrant activation of myogenic programs, potentially explaining increased off‐target cells rather than maturation upon prolonged culture. Czerniecki et al. used scRNA‐seq to demonstrate that kidney organoids generated via high‐throughput screening contained key nephron cell types but lacked collecting duct principal and intercalated cells, underscoring a current limitation in kidney organoid models [113].

Moreover, the ability of scRNA‐seq to resolve cellular heterogeneity makes it well‐suited for mapping differentiation trajectories and identifying transitional cell states during organoid development [108, 114]. For instance, Lindström et al. compared the transcriptional profiles of podocytes in hESC‐derived organoids with those in fetal kidneys to study podocyte differentiation states [115]. They found that loss of NPC markers corresponded to the onset of podocyte‐specific differentiation, thereby guiding further protocol optimisation. Furthermore, their scRNA‐seq data revealed species differences in gene expression regulation between human and mouse nephron and stromal progenitors [116], indicating potential limitations of cross‐species models for organoid assessment and application.

Building upon scRNA‐seq, multi‐omics approaches that integrate proteomics, metabolomics, and spatial transcriptomics have been applied to investigate kidney developmental trajectories and evaluate the fidelity of kidney organoids [117, 118, 119, 120]. Significantly, Liu et al. utilised high‐definition spatial RNA sequencing (hdST‐seq) to construct the first high‐resolution spatiotemporal transcriptomic atlas of the mouse kidney, analysing and revealing 40 migration‐related cell–cell communication events during kidney organogenesis [121]. Meanwhile, proteomics has been used to characterise the protein composition of kidney organoids, assessing their molecular relevance to human kidney disease models [119, 122, 123]. Metabolomic analysis broadens researchers' understanding of the relationship between the developmental microenvironment and phenotypic regulation [118, 124]. Moreover, in kidney organoid‐based fibrosis models, multi‐omics analyses have shown that inhibition of EZH2 attenuates TGF‐β1‐induced fibrotic gene expression and chromatin accessibility changes [125], highlighting a potential therapeutic strategy for renal fibrosis.

Taken together, the application of single‐cell and multi‐omics technologies has substantially refined the resolution at which kidney organoids can be evaluated, enabling detailed characterisation of cellular composition, differentiation states, and protocol‐dependent variability. These approaches provide a quantitative framework for assessing organoid fidelity in the context of renal development and disease modelling. When integrated with bioengineering platforms, they further facilitate the analysis of how engineered structural features and associated microenvironmental cues influence the continuum of cellular states, rather than merely altering terminal phenotypes. Such integrative strategies offer a basis for dissecting the sources of organoid heterogeneity and for guiding the optimisation of maturation and reproducibility.

## 6 Future Perspective

Driven by advances in differentiation protocols, bioengineering strategies, and single‐cell omics analyses, kidney organoids have made substantial progress and are gradually transitioning from conceptual models toward practical applications. For example, base‐edited PKD organoid models have been used as drug screening platforms to identify eukaryotic ribosome‐selective glycosides (ERSGs) as potential therapeutic candidates [11], underscoring their value in drug discovery. In terms of transplantation, ex vivo normothermic perfusion systems have been employed to deliver human kidney organoids into porcine kidneys [20], enabling in vivo evaluation of graft survival and immune responses.

Despite these advances, kidney organoids still face considerable hurdles before they can be applied in transplantation settings, with vascularisation and functional maturation being two of the most critical challenges. Although glomerulus‐like structures can form within organoids, they generally lack a well‐developed and stable capillary network [126, 127]. As a result, the supply of nutrients and oxygen within the organoid remains limited, which in turn constrains the formation of a functional glomerular filtration barrier and prevents the full recapitulation of essential renal functions. While in vivo transplantation and bioengineering strategies have partially addressed these limitations, achieving adult‐level renal function remains a major challenge. Recently, Przepiorski and Little et al. developed a strategy to reconstruct an endogenous vascular niche within an established kidney organoid bioreactor system [128, 129]. This approach resulted in kidney organoids with robust, integrated endothelial networks. Although the proportion of tubular cells, particularly distal tubular cells, was slightly reduced in vascularised organoids, this study provides a convenient strategy for building vascular niches during in vitro human kidney organogenesis.

With respect to maturity and functionality, constructing a kidney structure with meaningful physiological capacity requires not only the generation of all essential renal cell types but also the effective integration of a sufficient number of functionally mature nephrons into a unified collecting duct system [130]. Such structural and functional integration is critical for recapitulating renal physiology and remains one of the most formidable challenges in the field. A newly published study by McCracken et al. achieved nephron structures that were correctly polarised and connected to CD‐like structures for the first time in human organoids [131]. However, the growth and morphogenetic patterns of UB epithelial cells remained largely random, and their functional efficacy requires further investigation [131]. It is anticipated that future integration with more advanced bioengineering technologies will enable deeper resolution of kidney developmental molecular events, construction of more efficient and robust differentiation protocols, and simulation of appropriate developmental microenvironments. This will facilitate the translation of kidney organoids from bench to bedside.

**FIGURE 4:**
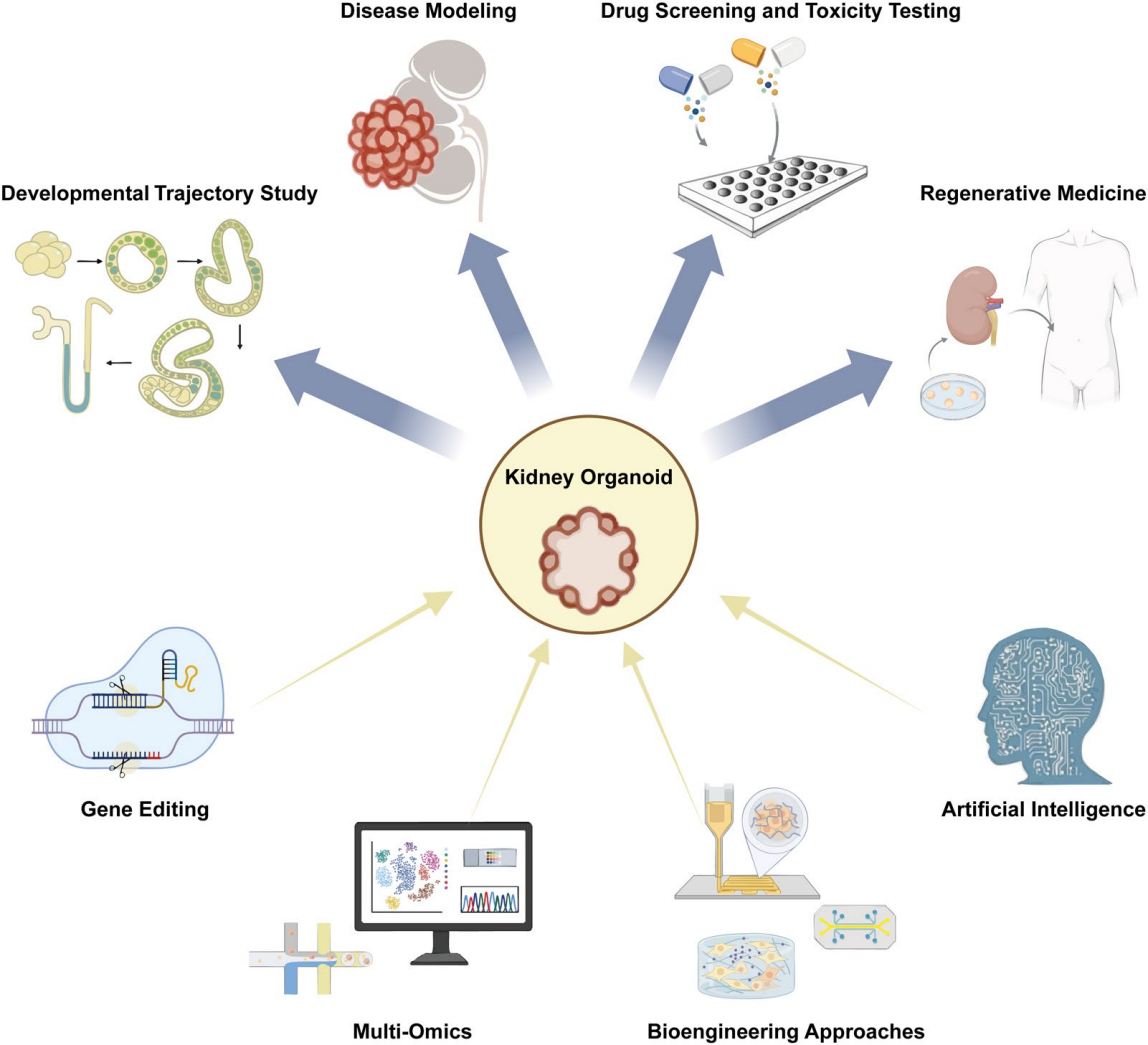
Future development and applications of kidney organoids. In the future, by integrating gene editing, bioengineering, and AI‐powered multi‐omics analyses, kidney organoids are poised to play a pivotal role across multiple fields. These include studying kidney development, disease modelling, drug screening and toxicity testing, and even regenerative medicine.

In addition, artificial intelligence (AI) is proving to be a powerful tool for uncovering kidney developmental mechanisms and accelerating drug discovery [132]. A recent study demonstrated that AI‐based drug discovery systems can improve hit rates by up to 17‐fold [133], highlighting their transformative computational and integrative capabilities in analysing large‐scale multi‐omics datasets derived from organoids [134]. Such advances may enable the identification of key regulatory factors governing kidney differentiation and help overcome current developmental bottlenecks. Moreover, integration with automated platforms may resolve standardisation challenges [135], thereby greatly enhancing the translational relevance of kidney organoids in preclinical research, drug screening, and therapeutic applications (Figure 4).

## 7 Conclusion

Kidney organoid technology is redefining the future landscape of renal disease research and clinical therapeutics. By recapitulating key developmental cues in vitro, kidney organoids have demonstrated remarkable potential in disease modelling, drug screening and nephrotoxicity evaluation, as well as in elucidating human kidney development, ultimately fostering advances toward personalised and precision medicine.

Nevertheless, significant challenges remain, including limited structural maturation, incomplete functional competence, insufficient vascularisation, and batch‐to‐batch variability. As discussed in this review, emerging bioengineering strategies, integration of multi‐omics technologies, and the incorporation of artificial intelligence and automated manufacturing platforms offer promising avenues for addressing these hurdles.

In brief, continued progress in understanding kidney development and refining differentiation and engineering methodologies is expected to yield more mature, robust, and functionally integrated kidney organoids. These advances will facilitate their translation from bench to bedside, bringing organoid‐based renal replacement and precision therapies closer to clinical reality.

## Author Contributions

**Bohong Guo:** writing – original draft preparation, visualisation, conceptualisation. **Jingyuan Zhang:** writing – review and editing, supervision, conceptualisation. **Xuening Fang:** writing – review and editing, conceptualisation. **Yangyang Liu:** writing – review and editing. **Yueyang Deng:** writing – review and editing. **Yuxin Xue:** writing – review and editing. **Zhengxi Wang:** writing – review and editing. **Xiaotao Dong:** supervision, project administration, resources, funding acquisition, conceptualisation. **Mengqi Jia:** supervision, resources. **Xiaodong Li:** resources, supervision.

## Funding

This work was supported by National Natural Science Foundation of China, 32100463; Natural Science Foundation of Guangdong Province, 2022A1515010642; China Postdoctoral Science Foundation, GZC20240403, 2024M750773; Science and Technology Department of Henan Province, 242102311191, 252102310322, 252102310145; Students' Innovation and Entrepreneurship Training Program of Henan University, XJ2025348.

## Conflicts of Interest

The authors declare no conflicts of interest.

## Data Availability

Data sharing is not applicable to this article as no datasets were generated or analysed during the current study.
